# Special Issue: Wildlife Microbiology

**DOI:** 10.3390/microorganisms9091968

**Published:** 2021-09-16

**Authors:** Kazunari Ushida, Richard Kock, Monica A. Sundset

**Affiliations:** 1 Department of Environmental Biology, Chubu University, Kasugai 487-8501, Japan; 2 The Royal Veterinary College, University of London, Hawkshead Lane, Hatfield AL9 7TA, UK; 3 Department of Arctic and Marine Biology, UiT—The Arctic University of Norway, 9037 Tromsø, Norway

Research on the effect of symbiotic microbes on the health of the host through their impact on digestion, the immune system, development, and behavior is accumulating. Host-associated communities can be disrupted by anthropogenic factors such as those that affect phenology (vegetation, life cycles, land-use, and climate), changing host pathogen dynamics (diseases), and environmental contamination. Seasonal and dietary factors also affect these microbial communities. The Anthropocene is characterized by dramatic and rapid systemic changes to global ecosystems, and a poorly understood aspect is the impact on microbial communities and balances between hosts and the myriad of organisms. It appears that emerging pathogens, in humans, are increasing relatively, and there is evidence this is not unique to the species. The need for microbiome research in an ecological context and as it applies to wildlife conservation is now urgent. The biodiversity of the host-associated microbiome should consequently be included as an essential component of wildlife management practices. The Special Issue “Wildlife Microbiology 2.0” of *Microorganisms* follows up on the Special Issue “Wildlife Microbiology” from 2018–2019. 

“Wildlife Microbiology 1.0” included seven papers on gut microbiology in a range of different animals and birds, including: the cecal microbiome in wild Japanese rock ptarmigans [1], the effect of season and diet on fiber digestion and ruminal bacterial community structure in muskoxen in Alaska [2], the fecal bacterial community and potential zoonotic bacteria of muskoxen in Greenland and Norway [3], the gut microbiota of the marsupial carnivorous Australian quoll [4], bacterial isolates from captive and wild mountain gorillas in Uganda [5], shifting gut microbiomes in captive-reared endangered voles [6], and adaptations to a frugivore/folivore diet in gorillas, chimpanzees, and wild forest elephants [7].

“Wildlife Microbiology 2.0” gathers twelve research papers related to the symbiotic gut microflora and bacterial pathogens in wildlife, and how these are affected by both natural and anthropogenic factors in their environment. This Special Issue includes studies of the oral microbial community of the micro-endemic and critically endangered admirable red-belly toad in southern Brazil [8], the gut microbiome of the invasive small Indian mongoose, a pervasive predator disrupting the native ecology in the Caribbean islands [9], the fecal microbiota of the Egyptian mongoose, a medium-size carnivore in Iberia, Europe [10,11], and the bacterial microbiome in the small intestine of hooded seals, a monogastric carnivore that goes through extreme fasting and re-feeding in early life [12]. The environment and wildlife species can also be reservoirs of human and animal pathogens and antibiotic resistance. However, even though direct infection and zoonosis are rare from wildlife, many new organisms that evolve and spread have roots in nature, with about 43% of the last half century of human-emerging infectious diseases acquired in some way from wildlife species origins [13]. This Special Issue includes studies of avian malaria in lovebirds hosted in an Italian zoo [14], *Mycobacterium bovis* infection in red foxes in areas with animal tuberculosis in France [15], the detection of wood mice carrying non-tuberculous Mycobacteria able to infect cattle and interfere with the diagnosis of bovine tuberculosis [16], and the phylogenetic relationship within the strongyloid nematode subfamily Phascolostrongylinae, a parasite found in the stomach and large intestine of Australian macropodid and vombatid marsupials [17]. It also includes studies of the antimicrobial activity of *Lactococcus lactis* subsp. *lactis* isolated from a Cuvier’s beaked whale stranded in Japan [18], studies of bacterial isolates and antimicrobial resistance in wildlife in Sicily in Southern Italy [19], and the characterization of multi-resistant ESBL-producing enterobacteria in fecal samples obtained from fruit bats in Gabon [20].

Altogether, these two Special Issues on Wildlife Microbiology present valuable data on the symbiotic gut microbiome, pathogens, and antimicrobial resistance in a range of animals and ecosystems not previously studied.

## References

[1] Ueda A., Kobayashi A., Tsuchida S., Yamada T., Murata K., Nakamura H., Ushida K. (2018). Cecal Microbiome Analyses on Wild Japanese Rock Ptarmigans (*Lagopus muta japonica*) Reveals High Level of Coexistence of Lactic Acid Bacteria and Lactate-Utilizing Bacteria. Microorganisms.

[2] Ungerfeld E.M., Leigh M.B., Forster R.J., Barboza P.S. (2018). Influence of Season and Diet on Fiber Digestion and Bacterial Community Structure in the Rumen of Muskoxen (*Ovibos moschatus*). Microorganisms.

[3] Andersen-Ranberg E.U., Barnes C.J., Rasmussen L., Salgado-Flores A., Grøndahl C., Mosbacher J.B., Hansen A.J., Sundset M.A., Schmidt N.M., Sonne C. (2018). A Comparative Study on the Faecal Bacterial Community and Potential Zoonotic Bacteria of Muskoxen (*Ovibos moschatus*) in Northeast Greenland, Northwest Greenland and Norway. Microorganisms.

[4] Burke C., Burnard D., Polkinghorne A., Webb J., Huston W.M. (2018). Cloacal and Ocular Microbiota of the Endangered Australian Northern Quoll. Microorganisms.

[5] Tsuchida S., Kakooza S., Nguema P.P.M., Wampande E.M., Ushida K. (2018). Characteristics of Gorilla-Specific Lactobacillus Isolated from Captive and Wild Gorillas. Microorganisms.

[6] Allan N., Knotts T.A., Pesapane R., Ramsey J.J., Castle S., Clifford D., Foley J. (2018). Conservation Implications of Shifting Gut Microbiomes in Captive-Reared Endangered Voles Intended for Reintroduction into the Wild. Microorganisms.

[7] Segawa T., Fukuchi S., Bodington D., Tsuchida S., Nguema P.P.M., Mori H., Ushida K. (2019). Genomic Analyses of *Bifidobacterium moukalabense* Reveal Adaptations to Frugivore/Folivore Feeding Behavior. Microorganisms.

[8] Mann M., Prichula J., de Castro Í., Severo J., Abadie M., Lima T.D.F., Caorsi V., Borges-Martins M., Frazzon J., Frazzon A. (2021). The Oral Bacterial Community in *Melanophryniscus admirabilis* (Admirable Red-Belly Toads): Implications for Conservation. Microorganisms.

[9] Becker A., Hill K., Butaye P. (2021). Unraveling the Gut Microbiome of the Invasive Small Indian Mongoose (*Urva auropunctata*) in the Caribbean. Microorganisms.

[10] Pereira A.C., Bandeira V., Fonseca C., Cunha M.V. (2020). Egyptian Mongoose (*Herpestes ichneumon*) Gut Microbiota: Taxonomical and Functional Differences across Sex and Age Classes. Microorganisms.

[11] Pereira A.C., Bandeira V., Fonseca C., Cunha M.V. (2020). Crosstalk Between Culturomics and Microbial Profiling of Egyptian Mongoose (*Herpestes ichneumon*) Gut Microbiome. Microorganisms.

[12] Acquarone M., Salgado-Flores A., Sundset M. (2020). The Bacterial Microbiome in the Small Intestine of Hooded Seals (*Cystophora cristata*). Microorganisms.

[13] IUCN (2021). Situation Analysis on the Roles and Risks of Wildlife in the Emergence of Human Infectious Diseases Publ.

[14] Cocumelli C., Iurescia M., Diaconu E., Galietta V., Raso C., Buccella C., Stravino F., Grande F., Fiorucci L., De Liberato C. (2021). *Plasmodium matutinum* Causing Avian Malaria in Lovebirds (*Agapornis roseicollis*) Hosted in an Italian Zoo. Microorganisms.

[15] Richomme C., Réveillaud E., Moyen J.-L., Sabatier P., De Cruz K., Michelet L., Boschiroli M.L. (2020). *Mycobacterium bovis* Infection in Red Foxes in Four Animal Tuberculosis Endemic Areas in France. Microorganisms.

[16] Varela-Castro L., Torrontegi O., Sevilla I.A., Barral M. (2020). Detection of Wood Mice (*Apodemus sylvaticus*) Carrying Non-Tuberculous Mycobacteria Able to Infect Cattle and Interfere with the Diagnosis of Bovine Tuberculosis. Microorganisms.

[17] Sukee T., Beveridge I., Sabir A.J., Jabbar A. (2020). Phylogenetic Relationships Within the Nematode Subfamily *Phascolostrongylinae* (Nematoda: *Strongyloidea*) from Australian Macropodid and Vombatid Marsupials. Microorganisms.

[18] Suzuki A., Suzuki M. (2021). Antimicrobial Activity of *Lactococcus lactis* subsp. *lactis* Isolated from a Stranded Cuvier’s Beaked Whale (*Ziphius cavirostris*) against Gram-Positive and -Negative Bacteria. Microorganisms.

[19] Gambino D., Vicari D., Vitale M., Schirò G., Mira F., Giglia M., Riccardi A., Gentile A., Giardina S., Carrozzo A. (2021). Study on Bacteria Isolates and Antimicrobial Resistance in Wildlife in Sicily, Southern Italy. Microorganisms.

[20] Nguema P.P.M., Onanga R., Atome G.R.N., Mbeang J.C.O., Mabika A.M., Yaro M., Lounnas M., Dumont Y., Zohra Z.F., Godreuil S. (2020). Characterization of ESBL-Producing Enterobacteria from Fruit Bats in an Unprotected Area of Makokou, Gabon. Microorganisms.

